# Identification of different myofiber types in pigs muscles and construction of regulatory networks

**DOI:** 10.1186/s12864-024-10271-9

**Published:** 2024-04-24

**Authors:** Chenchen Li, Yinuo Wang, Xiaohui Sun, Jinjin Yang, Yingchun Ren, Jinrui Jia, Gongshe Yang, Mingzhi Liao, Jianjun Jin, Xin’e Shi

**Affiliations:** 1 Laboratory of Animal Fat Deposition and Muscle Development, Key Laboratory of Animal Genetics, Breeding and Reproduction of Shaanxi Province, College of Animal Science and Technology, Northwest A&F University, Yangling, Shaanxi 712100 China; 2 Center of Bioinformatics, College of Life Sciences, Northwest A&F University, Yangling, Shaanxi 712100 China

**Keywords:** Pig, Muscle fiber type, miRNAs, mRNA, ceRNA network

## Abstract

**Background:**

Skeletal muscle is composed of muscle fibers with different physiological characteristics, which plays an important role in regulating skeletal muscle metabolism, movement and body homeostasis. The type of skeletal muscle fiber directly affects meat quality. However, the transcriptome and gene interactions between different types of muscle fibers are not well understood.

**Results:**

In this paper, we selected 180-days-old Large White pigs and found that *longissimus dorsi* (LD) muscle was dominated by fast-fermenting myofibrils and soleus (SOL) muscle was dominated by slow-oxidizing myofibrils by frozen sections and related mRNA and protein assays. Here, we selected LD muscle and SOL muscle for transcriptomic sequencing, and identified 312 differentially expressed mRNA (DEmRs), 30 differentially expressed miRNA (DEmiRs), 183 differentially expressed lncRNA (DElRs), and 3417 differentially expressed circRNA (DEcRs). The ceRNA network included ssc-miR-378, ssc-miR-378b-3p, ssc-miR-24-3p, XR_308817, XR_308823, SMIM8, MAVS and FOS as multiple core nodes that play important roles in muscle development. Moreover, we found that different members of the miR-10 family expressed differently in oxidized and glycolytic muscle fibers, among which miR-10a-5p was highly expressed in glycolytic muscle fibers (LD) and could target MYBPH gene mRNA. Therefore, we speculate that miR-10a-5p may be involved in the transformation of muscle fiber types by targeting the MYHBP gene. In addition, PPI analysis of differentially expressed mRNA genes showed that ACTC1, ACTG2 and ACTN2 gene had the highest node degree, suggesting that this gene may play a key role in the regulatory network of muscle fiber type determination.

**Conclusions:**

We can conclude that these genes play a key role in regulating muscle fiber type transformation. Our study provides transcriptomic profiles and ceRNA interaction networks for different muscle fiber types in pigs, providing reference for the transformation of pig muscle fiber types and the improvement of meat quality.

**Supplementary Information:**

The online version contains supplementary material available at 10.1186/s12864-024-10271-9.

## Introduction

Pork is an important component of the human diet. The indices for evaluating pork quality usually include meat color, pH, water level, flavor, tenderness, and marbling. Many factors can affect pork quality, such as genetics, nutrition, age, sex and breed. Muscle fibers are the basic units of muscle, and the proportion of muscle fibers determines meat quality. The physiological characteristics and metabolic patterns of muscle fibers are used to divide them into four categories: slow oxidizing (MyHC I, MYH7), fast oxidizing (MyHC IIa, MYH2), intermediate (MyHC IIx, MYH1) and fast enzymatic (MyHC IIb, MYH4). The metabolic types transition from oxidizing to enzymatic. Slow muscle fibers contain more myoglobin and less water within the muscle fibers, so muscles with a large proportion of slow muscle fibers are redder in color and drier in texture, while muscles with more fast muscle fibers are brighter, whiter and moister [1]. Thus, different types of muscle fibers influence meat quality traits such as tenderness, drip loss, and tethering power.

Peroxisome proliferator-activated receptor gamma coactivator 1α (PGC-1α) is a transcriptional coactivator that regulates mitochondrial biogenesis and respiratory function. Skeletal muscle-specific overexpression of PGC-1α induces fiber-type conversion through enhanced mitochondrial respiration and fatty acid oxidation in pigs [2]. With the development of RNA-seq, increasing evidence has shown that noncoding RNAs, including miRNAs, lncRNAs and circRNAs, are extensively involved in the regulation of skeletal muscle myofiber formation and myofiber type transformation. miRNA-27a promotes the transition of the porcine muscle fiber type from fast to slow muscle [3]. circMYLK4 significantly increases the mRNA and protein levels of slow muscle marker genes [4]. Recent studies have shown that the ceRNA regulatory network with a core of miRNAs has an important role in myofiber formation. Ju constructed a ceRNA network analysis of potential regulatory lncRNAs and circRNAs in chicken oxidative and glycolytic muscle fibers [5]. The lncRNA MyHC IIA/X-AS acts as a competing endogenous RNA that sponges microRNA-130b (miR-130b) and thereby maintains MyHC IIx expression and the fast fiber type [6]. Through high-throughput sequencing of longissimus miRNAs, lncRNAs, and circRNAs from of Landrace and Lantang pigs, and the results showed that 40 circRNAs participated in sponge modulation of 26 miRNA-mediated ceRNA interactions [7]. Circular RNA screening identified circMYLK4 as a regulator of fast/slow myofibers in porcine skeletal muscle [4], and circMYLK competitively binds to miR-29a and abrogates the inhibitory effects of the miR-29a VEGFA/VEGFR2 and downstream Ras/ERK signaling pathways [8]. These findings suggested that miRNAs play key nodes in the ceRNA regulatory network. However, the role of the miRNA regulatory network in the regulation of porcine muscle fiber types has been less studied, and key miRNAs need to be further identified.

In this study, we conducted full transcriptome sequencing on *longiscus dorsi* (LD) muscle and soleus (SOL) muscle tissues of 180-days-old large white pigs, and identified 312 differentially expressed mRNAs (DEmRs), 30 differentially expressed miRNAs (DEmiRs), 183 differentially expressed lncRNAs (DElRs) and 3417 differentially expressed circRNAs (DEcRs). The ceRNA network revealed that ssc-miR-378, ssc-miR-378b-3p, ssc-miR-24-3p, XR_308817, XR_308823, SMIM8, MAVS and FOS are core nodes that play important roles in muscle development. We also screened out that the miR-10 family, ACTC1, ACTG2 and ACTN2 genes may be involved in regulating the type transformation of porcine muscle fibers. In this study, the key regulatory molecules that may regulate meat quality traits were screened to provide a theoretical basis for improving the quality of livestock and poultry products.

## Materials and methods

### Experimental animals

The study was carried out under the supervision of the Experimental Animal Ethics Committee of Northwest A&F University (Yangling, Shaanxi Province, China, No. NWAFU-20,220,108). Large white pigs (180-days-old) were obtained from the Northwest A&F University Experiment Station (Yangling, Shaanxi, China). Large white pigs (*n* = 4) were fasted for 24 h before slaughter but had free access to drinking water. The pigs were euthanized using electrical stunning and bled. Immediately after slaughter, samples were taken from the middle part of the SOL and LD, three portions were taken per pig, the samples were frozen in liquid nitrogen, and the samples were stored at − 80 °C for further use. The SOL and LD regions of a large white pig were selected for transcriptome sequencing.

### Quantitative real-time PCR

Total RNA was extracted from the tissues and cells. In brief, 0.5 g of tissue sample was ground with 1 ml of TRIzol and magnetic beads. Trichloromethane (200 ul) was then added. The homogenate was centrifuged for 15 min. Supernatant was then mixed with an equal volume of isopentane, followed by a centrifugation for 20 min. The pellet was collected and resuspended with DEPC water to dissolve the RNA. RNA samples were reverse-transcribed using a standard system (Vazyme, R323-01). For U6, miR-99b, miR-125a, miR-378, miR-10a-3p, miR-10a-5p and miR-24-3p detection, a system with specific RT primers and specific reverse primers was used according to the manufacturer’s instructions (Vazyme, R312-02). qRT‒PCR was performed with a Bio-iQ5 real-time PCR system (Bio-Rad, Hercules, CA, USA) using a one-step SYBR PrimeScript RT‒PCR kit (TaKaRa, Otsu, Japan). The expression levels of the genes of interest were normalized to those of the reference genes 18 S RNA and U6, and the relative expression was calculated using the ΔΔCt method. The sequences of primers used for qRT‒PCR were as follows:

**Table:** 

Oligo	Sequences (5’—3’)	Tm(℃)
18 S RNA	F: CCCACGGAATCGAGAAAGAG	60
	R: TTGACGGAAGGGCACCA	
MYH7	F: AAGGGCTTGAACGAGGAGTAGA	60
	R: TTATTCTGCTTCCTCCAAAGGG	
MYH2	F: GCTGAGCGAGCTGAAATCC	60
	R: ACTGAGACACCAGAGCTTCT	
MYH1	F: AGAAGATCAACTGAGTGAACT	60
	R: AGAGCTGAGAAACTAACGTG	
MYH4	F: ATGAAGAGAGGAACCACATTA	60
	R: TTATTGCCTCAGTAGCTTG	
chr2:17755529–17,771,066	F: GCTCAAATGAAGCAGGATCCAC	60
	R: TGCAGCAGTTTTGGGGAAAC	
chr8: 4,258,670–4,260,865	F: GGGATGACCGCTCCTACAAG	60
	R: CCGGTCGATGTCGTAGATGG	
XR_305855.1	F: TGAAGGACGGCTTACCCTTG	60
	R: AAGGGCGGAAAGCTGTAAGG	
XR_309093.1	F: AGCAACAGATCGTCACTCGG	60
	R: GTGGTGGGTGGTAGGAGTTG	
HOXA10	F: TGGCCTTTGTTCGCTTCTGA	60
	R: AATCACTGCCAAGGGAGAGC	
ZIC1	F: AGTCCCCGTTCAGAGCACTA	60
	R: GGCTCGATCCACTTGCAGAT	

The U6, miR-99b, miR-125a, miR-378, miR-10a-3p, miR-10a-5p and miR-24-3p primers were purchased from RiboBio (Guangzhou, China)

### Western blotting

Muscle samples were lysed using RIPA buffer (Yeasen, Shanghai, China) containing a mixture of protease and phosphatase inhibitors. After centrifugation (12,000 rpm, 10 min, 4 °C), proteins were separated on 10% polyacrylamide gels and transferred to polyvinylidene difluoride membranes (Millipore, IPVH00010). During the blotting process, the polyvinylidene fluoride membrane is sheared before hybridization with the antibody. The membranes were incubated with the primary antibody overnight at 4 °C followed by the secondary antibody for 1 h at room temperature and visualized using the Image Lab system. The densities of the protein bands were quantified using ImageJ software. We used antibodies against GAPDH (Proteintech, 60004-1-lg), MYH7 (Proteintech, 22280-1-AP), MYH2 (Proteintech, 55063-1-AP), MYH1 (Proteintech, 67299-1-AP), and MYH4 (Proteintech, 20140-1-AP), and HRP-conjugated goat anti-mouse IgG or goat anti-rabbit IgG secondary antibodies (BOSTER, China).

### Tissue section staining

For tissue immunofluorescence staining, fresh LD and SOL muscles were transferred to an OCT embedding agent, embedded in isopentane, and frozen in liquid nitrogen; then 20 μm muscle samples were cut using a frozen microtome (Sakura, Japan). The sections were incubated with anti-MYHC7, anti-MYHC2, and anti-MYHC4 primary antibodies overnight and subsequently with a fluorophore-conjugated secondary antibody for 1 h; the sections were subsequently photographed using cellSens software (Olympus Corporation). We used antibodies against MYH7 (DSHB, BA-D5), MYH2 (DSHB, SC-71), and MYH4 (DSHB, 10F5), together with Alexa Fluor 594-conjugated goat anti-mouse IgM (Immunoway, RS3609), cross-adsorbed goat anti-mouse IgG1 (Thermo Fisher, A-21,121), and cross-adsorbed goat anti-mouse IgG2b (Thermo Fisher, A-21,140) secondary antibodies.

### RNA sequencing

Isolation and purification of total RNA from samples using Trizol reagent, Secondly, we purified and sequenced the RNA using the Illumina NovaSeq™ 2500 system (LC-BIO, China) paired-end RNA-seq method. RNA-seq was performed by RiboBio (Guangzhou, China). The raw image data files produced by high-throughput sequencing were transformed into raw sequencing sequence data (RawData) by CASAVA/Basecall_T7_GPU_1.2.0.26_Centos Base Calling analysis, and the reference genome and gene model annotation files were downloaded from the genome website (Sscrofa11.1, GCA_000003025.6) and used for de novo gene prediction in StringTie (2.1.2). Novel genes were sequenced against the NR, Swiss-Prot, GO, COG, KOG, Pfam, and KEGG databases using BLAST (2.2.31) software. Quantitative gene expression analysis was performed using StringTie software. For samples with biological duplicates, differential expression analysis between sample sets was performed using DEseq2 (1.26.0) software to obtain the set of differentially expressed genes between the two biological conditions. The Q30 quality threshold (Phred quality score > 30) was met for 95.4% of the data. The sample and sequencing data generated in this study were of good quality and were reliable for subsequent bioinformatic analysis. And the amount of sequencing data (clean reads) per library was about 6 Gb.

### Cell culture

Human embryonic kidney 293T cells (Shanghai Branch Cell Bank of the Chinese Academy of Sciences) were cultured in high glucose Dulbecco’s modified Eagle’s medium (DMEM, Servicebio, G4510) supplemented with 10% foetal bovine serum (ZETA LIFE, Z7186FBS-500) and 100 IU/mL penicillin‒streptomycin at 5% CO_2_ and 37 °C.

### Dual luciferase reporter assays

The 3’-UTR of SMIM8, MAVS and MYBPH was synthesized by Tsingke Biotechnology Co. (Beijing, China). Human embryonic kidney 293T cells were seeded at 15,000 cells per well in a 24 well culture plate, and 250 ng of psiCHECK2-SMIM8-3’-UTR and psiCHECK2-MAVS-3’-UTR was cotransfected with 50 nM of either miR-24-3p mimics or NC, and 250 ng of psiCHECK2-MYBPH-3’-UTR was cotransfected with 50 nM of either miR-10a-5p mimics or NC when the cells reached 70% confluence. After transfection for 48 h, the relative luciferase activities of Renilla compared with those of firefly were measured with a Dual-Luciferase reporter assay system (Promega, E1910) according to the manufacturer’s protocol (US EVERBRIGHT, Suzhou, China) [9].

### Differential expression analysis

The “DEGseq” package in R software was used to identify DEmRs, DEmiRs, DElRs and DEcRs with the thresholds listed in Table 1.

**Table 1:** Thresholds for in differential expression analysis

LD vs. SOL	Threshold	
	|log2 fold change|	adj. pvalue
mRNA	1	0.05
miRNA	0.5	0.05
lncRNA	2	0.05
circRNA	2	0.05

### Prediction of target genes

PITA [10], RNA22 [11], and TargetScan [12] were utilized to predict the potential candidate miRNAs that can target DEmRs. miRNAs predicted by all three software programs were identified as the prediction DEmiRs. Only miRNAs found from miRNA differential analysis and predicted by targeting differentially expressed mRNAs (predicted DEmiRs) were screened out for ceRNA network construction. The three algorithms were subsequently used to predict the target pairs of the miRNAs mentioned above, DEmRs and DElRs. Afterward, the ceRNA network was constructed and visualized by Cytoscape v3.9.1 [13].

### Functional enrichment analysis

To gain an in-depth understanding of the biological importance of the DEmRs and the target genes of the miRNAs, respectively, we performed Gene Ontology (GO) and Kyoto Encyclopedia of Genes and Genomes (KEGG) pathway enrichment analyses via DAVID [14]. A p-value < 0.1 was selected as the cutoff criterion for GO term and KEGG pathway enrichment analysis.

### PPI network construction and analysis of modules

In this study, previously identified DEmRs were mapped to the STRING database to analyze protein‒protein interaction (PPI) networks. A combined score ≥ 0.4 was set as the threshold, and *Sus scrofa* was chosen as the organism. The transcription factor information was acquired from Animal TFDB v3.0 [15]. In addition, Cytoscape v3.9.1 [13] was utilized to construct the PPI network. The Cytoscape plugin Molecular Complex Detection (MCODE) v2.0.2 [16] was utilized to construct subnetworks with a degree cutoff = 2, maximum = 100, a node score cutoff = 0.2 and a kcore = 2.

### Genes associated with diseases and traits

Using publicly available data from the Online Mendelian Inheritance in Animals (OMIA) [17], we obtained the DEmRs in which mutations have been shown to result in Mendelian traits in *S. scrofa* and other animals in Artiodactyla (*Bos taurus*, *Ovis aries*). Additionally, sequence collection and alignment were performed using NCBI, Ensembl and DNAMAN software. The ISwine database [18] was used for collecting the QTXs of the key genes. For QTX, we focused only on those related to muscles and some traits with economic benefit (disease, fat, growth, meat quality, reproduction and slaughter).

### Statistical analysis

Graphs were generated using GraphPad Prism 8.02. Data are the means ± SEMs. The significance of between-group differences was assessed using a Student’s *t* test or one- or two-way analysis of variance (*P < 0.05; **P < 0.01).

## Results

### The difference between LD and SOL muscles

**Fig. 1:**
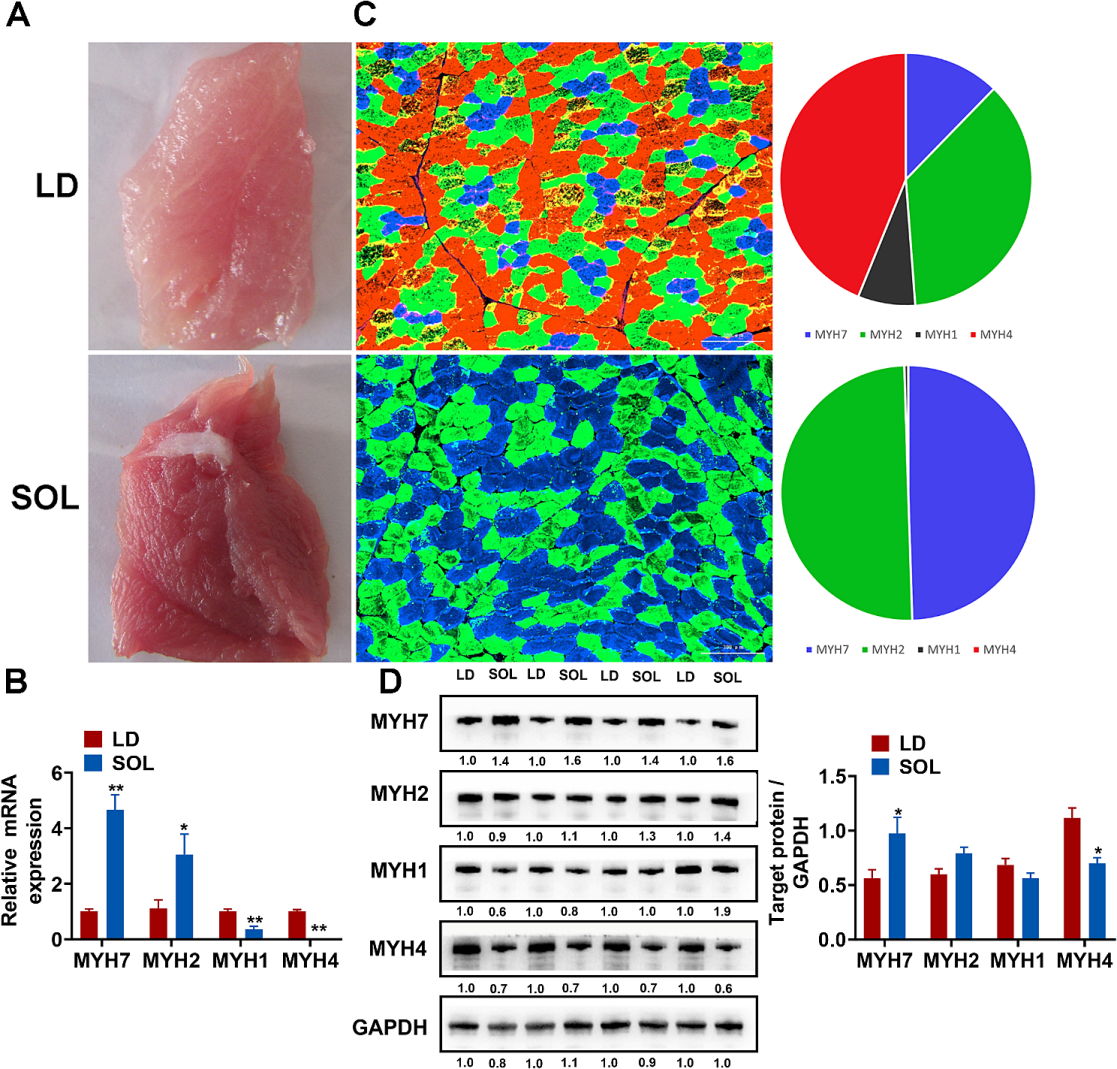
Identification of the differences between LD and SOL muscles. (A) Photographs of the LD and SOL muscles. (B) The mRNA expression levels of *MYH7*, *MYH2*, *MYH1*, and *MYH4* in LD and SOL muscles were determined via qRT‒PCR (*n* = 4). (C) The fiber types in the LD and SOL muscles were stained and enumerated. MYH7, MyH2, and MyH4 were used as markers of type I, type IIa, and type IIb fibers, respectively (*n* = 4). (D) Protein levels of MYH7, MYH2, MYH1, and MYH4 were measured via Western blotting in the LD and SOL muscles (*n* = 4). The results are presented as the means ± SEMs. The significance of differences between means was determined via an independent sample *t* test (*P < 0.05; **P < 0.01)

LD and SOL are representative of glycolic muscle and oxidized muscle respectively, and their functions differ depending on the main metabolic mode. These muscle types differ not only in their functions, but also in their internal gene expression. We isolated the LD and SOL muscles and found that, compared with the LD muscle, the SOL had a redder flesh color, a greater myoglobin content, and a drier surface (Fig. 1A). The expression of *MYH7, MYH2, MYH1* and *MYH4* determines the main metabolic pattern. Using RT‒qPCR, we found that the expression of the *MYH7* and *MYH2* genes was significantly greater in the SOL group than in the LD group. In contrast, the expression of the MYH1 and MYH4 genes was significantly greater in LD than in SOL (Fig. 1B). The frozen section results showed that MYH4 and MYH2 accounted for the majority of the LD muscle, which was dominated by enzymatic muscle fibers. Moreover, the SOL consisted of two main muscle fibers, MYH7 and MYH4, which were predominantly oxidative (Fig. 1C). Similarly, Western blot analysis revealed that the protein expression levels of MYH4 and MYH1 were significantly greater in the LD group than in the SOL group. In contrast, the protein levels of MYH7 and MYH2 were significantly lower in LD than in SOL (Fig. 1D), which was consistent with the mRNA levels.

### Construction and analysis of the ceRNA network

**Fig. 2:**
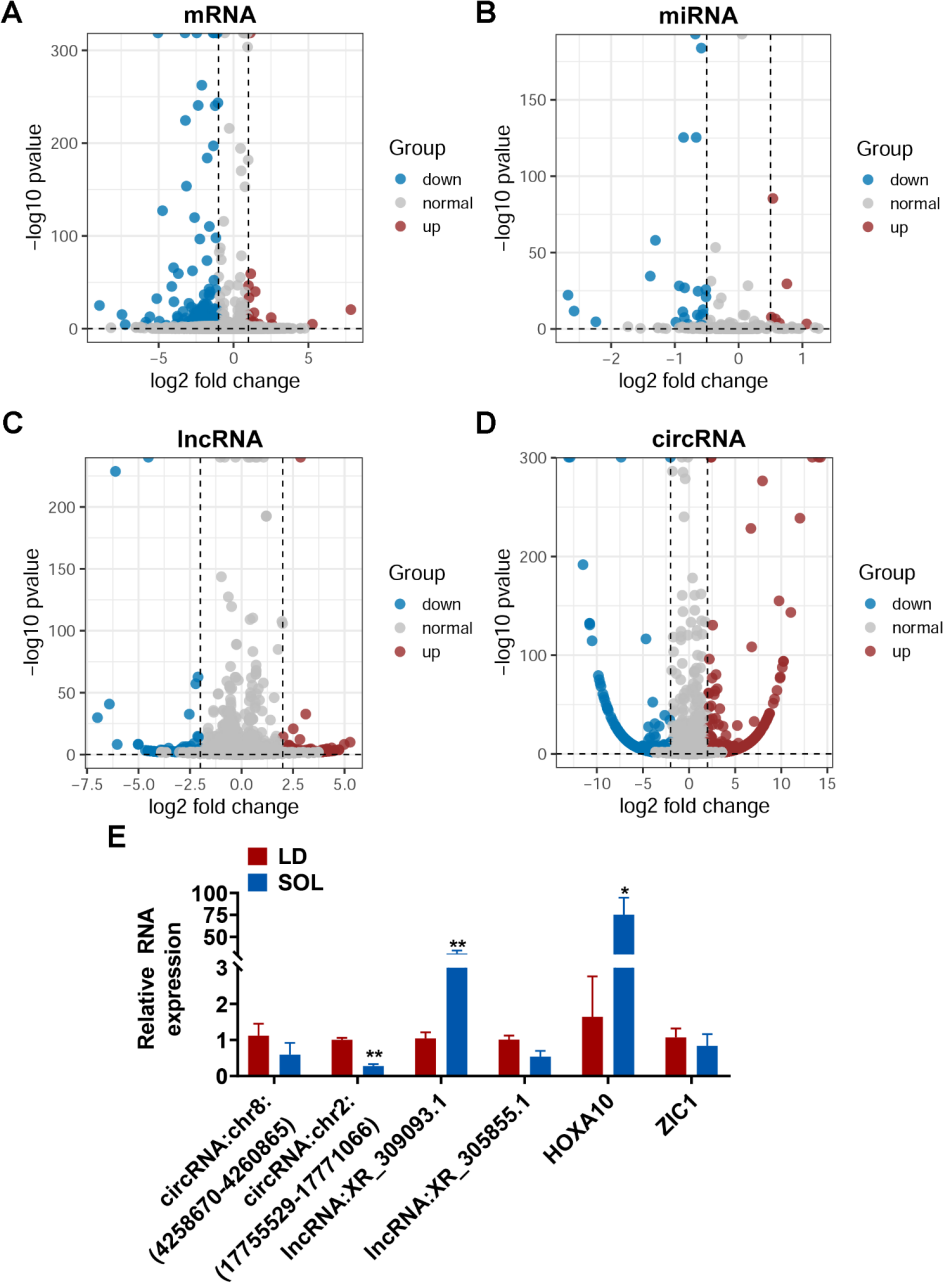
Differential expression analysis and screening of the miRNAs for ceRNA network construction. (A-D) Volcano plot showing the differential expression of mRNAs, miRNAs, lncRNAs, anf circRNAs in LD versus SOL. E. Expression levels of circRNAs, lncRNAs and mRNAs in the LD and SOL muscles (*n* = 4). The data are presented as log2-fold changes and log10 p values

We performed transcriptome sequencing of the LD and SOL muscles. According to the abovementioned criteria, 312 DEmRs, 30 DEmiRs, 183 DElRs and 3,417 DEcRs were ultimately screened out in LD versus SOL (Fig. 2A-D). In order to verify the accuracy of RNA-seq, we used RT-qPCR to verify 6 randomly selected differentially expressed genes, and the results showed that RT-qPCR was consistent with RNA-seq (Fig. 2E).

**Fig. 3:**
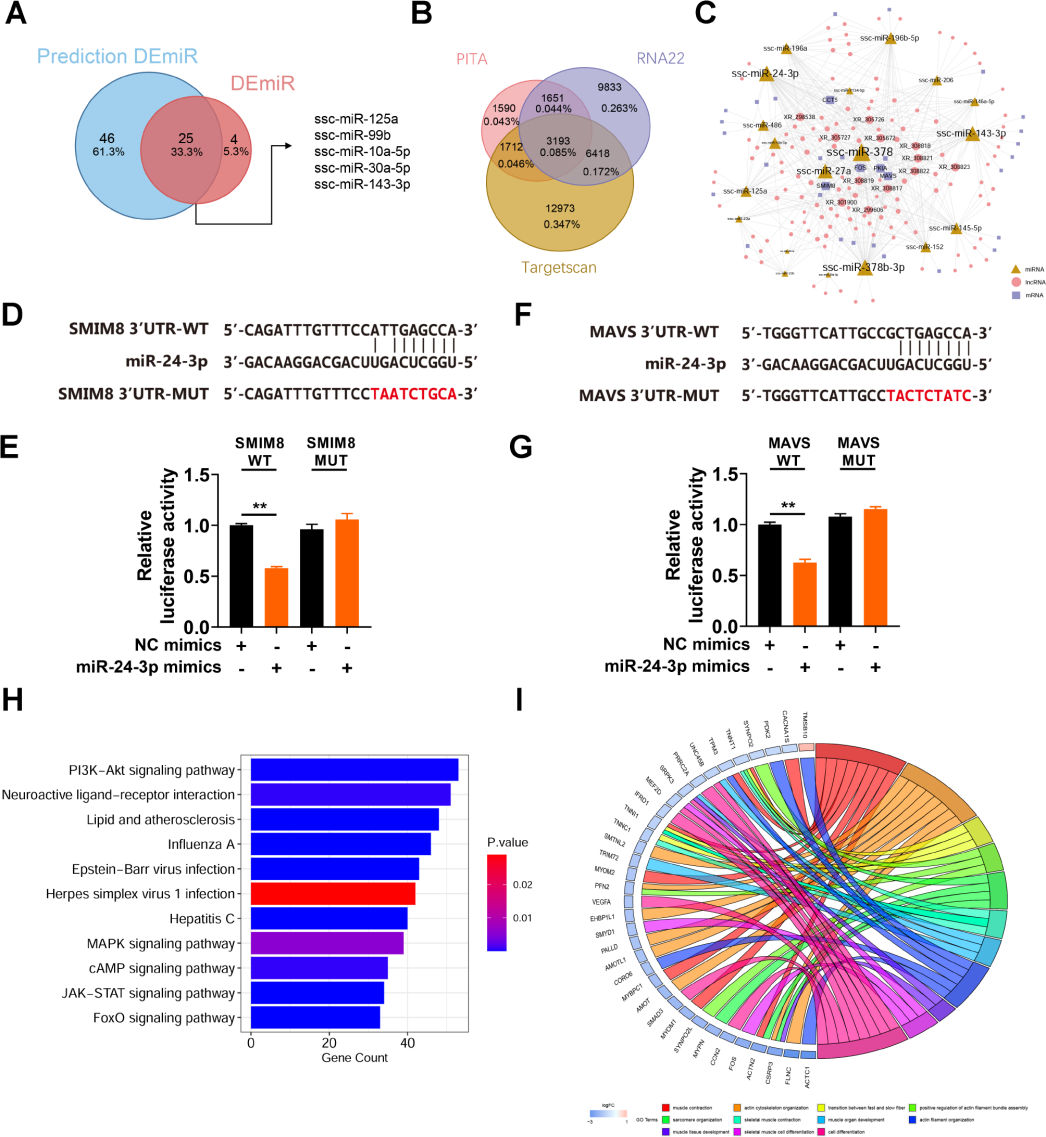
ceRNA network construction and functional enrichment analysis of the target genes. (A) Venn diagram showing the overlap of DEmiRs identified by small RNAseq quantification and RNA seq-based prediction. (B) Target gene prediction of 25 miRNAs. (C) The lncRNA‒miRNA‒mRNA ceRNA network. Yellow triangles, pink circles and purple squares indicate mRNAs, lncRNAs and mRNAs, respectively. The node size reflects the degree of the same kind of molecule. (D) Dual-luciferase reporter assay of wild-type (WT) plasmids and mutantion (MUT) plasmids. Sequence alignment of miR-24-3p with the SMIM8 WT sequence and MUT sequence. (E) Relative Renilla luciferase activity analysis after transfection with NC mimics + SMIM8 WT plasmids, miR-24-3p mimics + SMIM8 WT plasmids, NC mimics + SMIM8 MUT plasmids, and miR-24-3p mimics + SMIM8 MUT plasmids (*n* = 6). (F) Dual-luciferase reporter assay of WT sequence and MUT plasmids. Sequence alignment of miR-24-3p with the MAVS WT sequence and MUT sequence. (G) Relative Renilla luciferase activity analysis after transfection with NC mimics + MAVS WT plasmids, miR-24-3p mimics + MAVS WT plasmids, NC mimics + MAVS MUT plasmids, and miR-24-3p mimics + MAVS MUT plasmids (*n* = 6). (H) Bar plot of the results of KEGG enrichment analysis of the target genes. (I) Chord plot of the targeted genes and associated Gene Ontology pathways

PITA, RNA22, and TargetScan were utilized to predict the potential candidate miRNAs that can target DEmRs. The overlaps among these algorithms were identified as the predicted DEmiRs. There were 71 predicted DEmiRs, 25 of which were DEmiRs identified by small RNA-seq (Fig. 3A). Subsequently, we used three prediction algorithms to analyze the differentially expressed mRNAs and lncRNAs with binding potential of these 25 miRNAs (Fig. 3B). After screening for differentially expressed genes, we constructed a ceRNA regulatory network consisting of 19 miRNAs, 32 mRNAs, and 134 lncRNAs (Fig. 3C). In the ceRNA regulatory network, we found that 3 miRNAs (ssc-miR-378, ssc-miR-378b-3p and ssc-miR-24-3p), 2 lncRNAs (XR_308817 and XR_308823) and 3 mRNAs (SMIM8, MAVS and FOS) had higher node degrees than the other miRNAs, indicating that they were at the core of the network and may have important biological functions in LD and SOL (Fig. 3C). Studies have shown that miR-24-3p can promote myoblast differentiation and regeneration [19], so we selected miR-24-3p for ceRNA verification. We predicted the presence of miR-24-3p binding sites on the mRNA sequences of SMIM8 and MAVS genes, so we conducted dual luciferase activity reporting experiments. The results showed that miR-24-3p could specifically target the mRNA of SMIM8 and MAVS genes, while mutating the miR-24-3p binding site could eliminate the inhibitory effect of miR-24-3p on the target SMIM8 and MAVS genes (Fig. 3D-G). The target genes were significantly enriched in signaling pathways related to muscle and skeletal muscle growth and development (the PI3K-Akt, MAPK, JAK-STAT, and FoxO signaling pathways) (Fig. 3H), and these signaling pathways are related to differences in muscle fiber differentiation and energy metabolism. According to the GO enrichment results, several genes (TNNT1, TNNC1, SRPK3, etc.) were enriched in biological processes related to muscle fibers and muscle contraction (Fig. 3I).

### The expression of the miR-10 family in LD and SOL

**Fig. 4:**
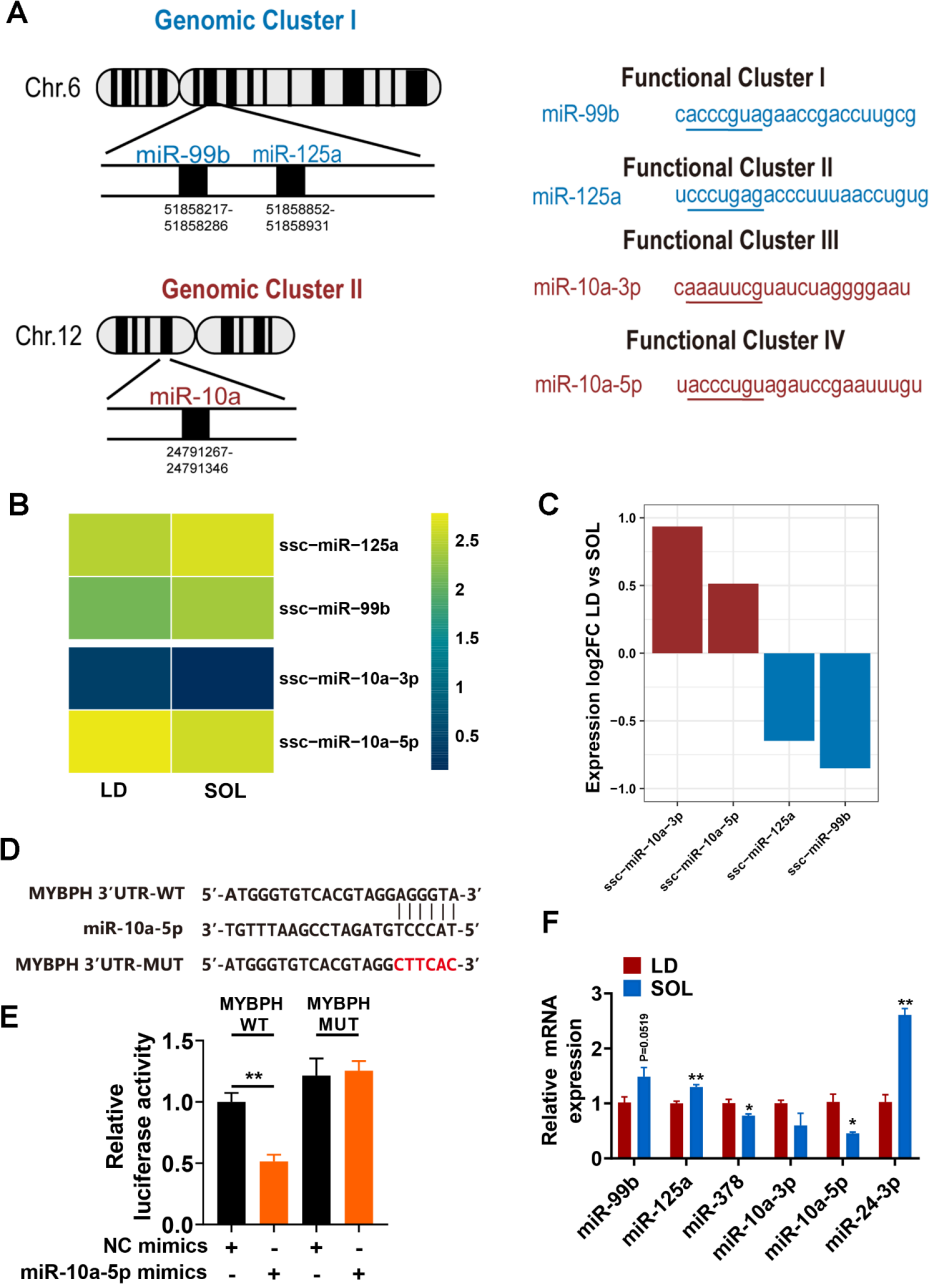
Expression of the miR-10 family in LD and SOL. (A) Schematic representation of the mir-10 family in *S. scrofa*. The miRNAs of the miR-10 family were grouped into two distinct genomic clusters based on different genomic locations and four functional clusters based on seed sequence similarities. (B) Heatmap of miR-10 family expression in LD and SOL cells. The data are presented as lgRPM values extracted from the small RNA-seq dataset. (C) Differential expression of miR-10 cluster-associated miRNAs in LD and SOL samples. (D) Dual-luciferase reporter assay of WT plasmids and MUT plasmids. Sequence alignment of miR-10a-5p with the MYBPH WT sequence and MUT sequence. (E) Relative Renilla luciferase activity analysis after transfection with NC mimics + MYBPH WT plasmids, miR-10a-5p mimics + MYBPH WT plasmids, NC mimics + MYBPH MUT plasmids, and miR-10a-5p mimics + MYBPH MUT plasmids (*n* = 6). (F) Expression levels of miR-99b, miR-125a, miR-378, miR-10a-3p, miR-10a-5p and miR-24-3p in the LD and SOL muscles (*n* = 4). The results are presented as the means ± SEMs. The significance of differences between means was analyzed via an independent sample *t* test (*P < 0.05; **P < 0.01)

There were 71 predictd DEmiRs, 25 of which were DEmiRs identified by small RNA-seq (Fig. 2E). Interestingly, 3 of the 25 miRNAs, miR-10a-3p, miR-99b, and miR-125a, belonged to the miR-10 family. We further characterized the expression of the miR-10 family in LD and SOL. The miR-10 family comprises seven miRNA members in pigs, namely, miR-10a, miR-10b, miR-99a, miR-99b, miR-100, miR-125a, and miR-125b. Based on their location in the genome, miR-99 and miR-125a were grouped into genomic cluster I, whereas genomic cluster II was composed of miR-10a (Fig. 4A). Functionally, these miRNAs target four different sequences and are divided into different functional groups. We observed that the expression of miR-10 genomic cluster I (miR-125a, miR-99b) was greater in the SOL group. In contrast, the expression of the genomic cluster II members miR-10a-3p and miR-10a-5p was greater in the LD group than in the SOL group (Fig. 4B-C). We speculated that the miR-10 family is tightly regulated by muscle fiber transformation. The target genes of the miR-10 family in the ceRNA network included DES [20], MYBPH [21] and SMAD3 [22]. To verify this hypothesis, we constructed MYBPH wild-type (WT) plasmids and mutantion (MUT) plasmids for co-transfection with miR-10a-5p mimics, and dual luciferase reporter genes determined that miR-10a-5p and MYBPH had strong binding ability (Fig. 4D-E). We used RT‒qPCR to show that miR-125a and miR-24-3p had significantly greater expression in LD than in SOL, whereas miR-378 and miR-10a-5p had significantly greater expression in SOL than in LD, which was consistent with the predicted results (Fig. 4F).

### Target gene function analysis of high-abundance miRNAs

**Fig. 5:**
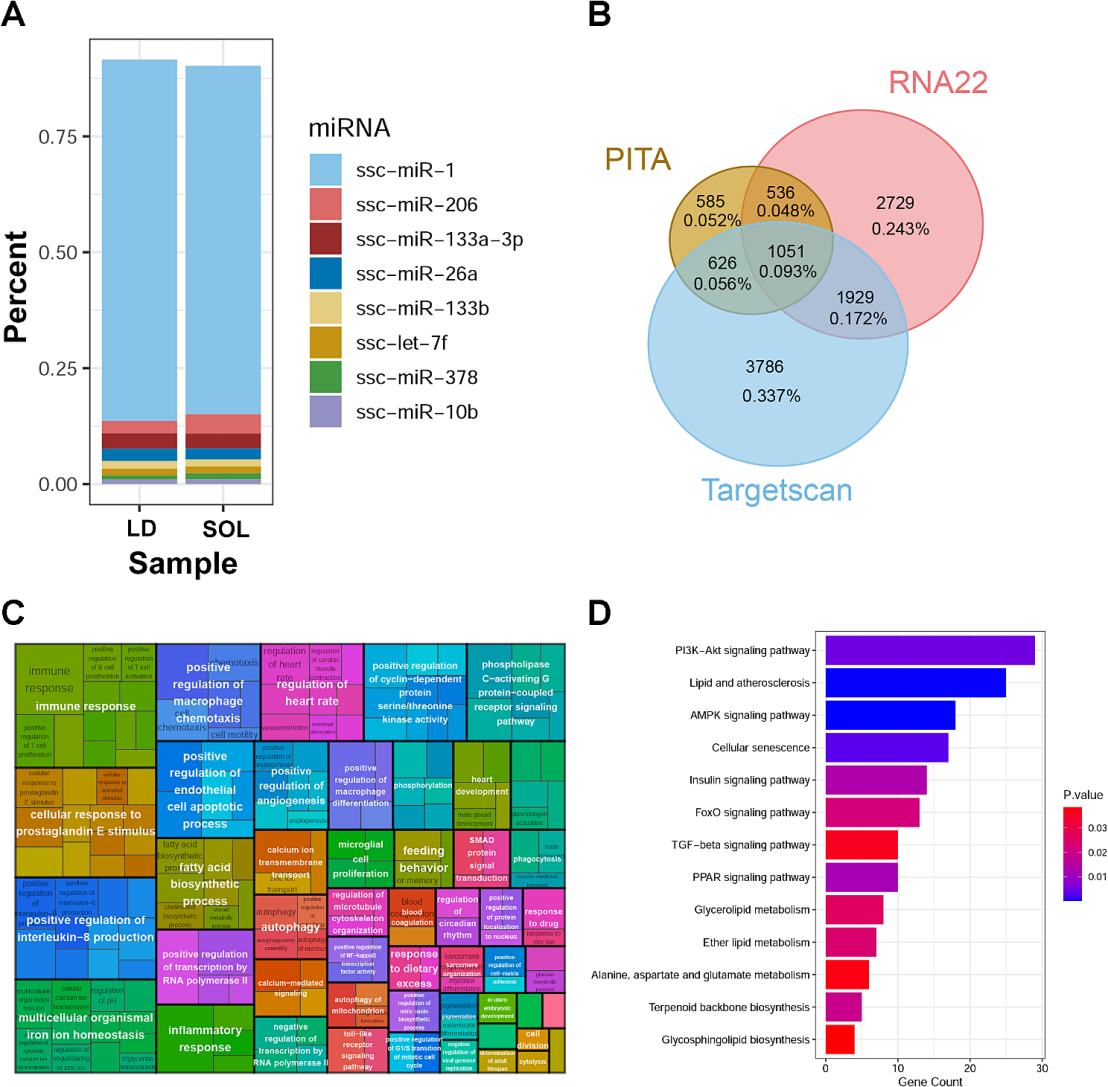
Target gene prediction and functional enrichment analysis of high-abundance expressed miRNAs in LD and SOL. (A) The top 8 miRNAs with the highest expression in LD and SOL. (B) Target gene prediction of 8 high-abundance miRNAs. (C) GO enrichment analysis of target genes. (D) KEGG enrichment analysis of target genes

The top 8 miRNAs with the highest expression in LD and SOL were consistent and accounted for more than 90% of the total miRNAs (Fig. 5A). Target gene prediction was performed for these 8 miRNAs (Fig. 5B), and GO and KEGG enrichment analyses of the target genes were performed. The GO enrichment results were related mainly to the positive regulation of the immune response, cholesterol metabolic process, multicellular organismal iron ion homeostasis and other biological processes (Fig. 5C). KEGG pathway enrichment revealed that the target genes were significantly enriched in muscle development related signaling pathways (PI3K-Akt, AMPK, FoxO, and the TGF-beta signaling pathway) and metabolic synthesis processes (glycerolipid, ether lipid metabolism, terpenoid backbone, and glycosphingolipid biosynthesis) (Fig. 5D), indicating that genes targeted by high-abundance miRNAs regulate muscle growth and energy metabolism primarily through the above biological processes.

### Analysis of the PPI network and modules

**Fig. 6:**
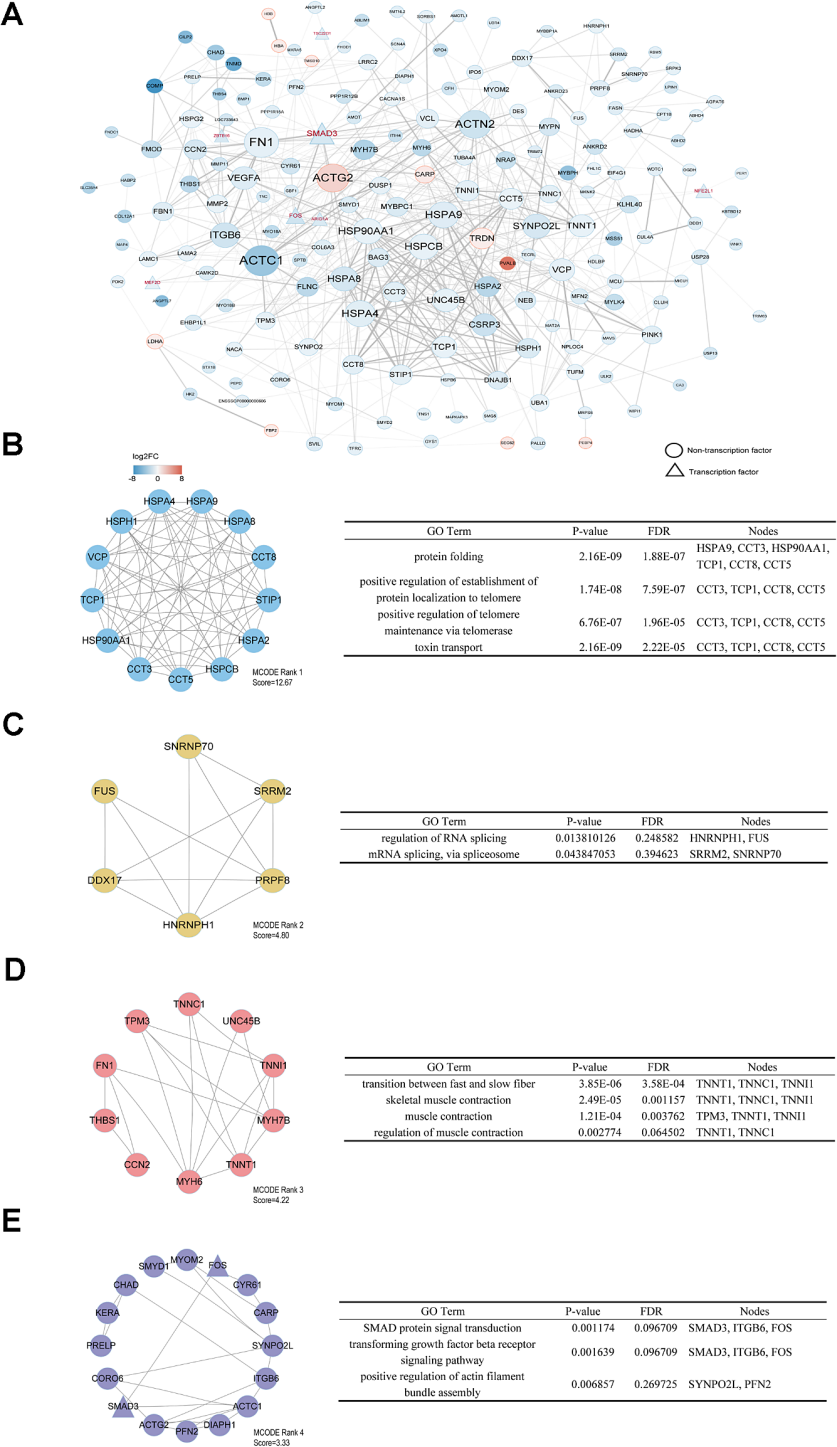
Analysis of the PPI network and modules. (A) Cytoscape software was used to construct protein‒protein interaction networks. Transcription factors are represented with triangles. The color represents the log2FC. (B-E) The top 4 modules from the protein–protein interaction network and the enriched pathways of the modules

We input the DEmRs into the STRING database. The nodes with high degrees were ACTC1, FN1, ACTG2, ACTN2, HSPA4, HSPA9, HSPCB, HSP90AA1, and ITGB6 (Fig. 6A). Among these genes, ACTC1 had the highest node degree, which was 31. ATAC1 influences the contractile properties of smooth muscle [23]. Several nodes with high absolute values of log_2_FC were COMP, TNMD and PVALB. Moreover, 181 nodes and 621 edges were analyzed using the MCODE plug-in. The top 4 significant modules were selected, and the functional annotation of the genes involved in the modules was analyzed (Fig. 6B-E). Enrichment analysis revealed that the genes in module 3 were mainly associated with transitions between fast and slow fibers, skeletal muscle contraction, and muscle contraction. These findings suggested that these newly identified high node genes are involved in muscle fiber formation and muscle fiber type transformation.

### Genes associated with disorders and traits

We further explored the relationship between DEmRs and Mendelian inheritance. Using publicly available data from the OMIA, we obtained the DEmRs in which mutations have been shown to result in Mendelian traits in pigs and other animals in Artiodactyla (cattle and sheep) (Table 2). We observed that MYH7, RYR1, FBN1, TNNT1, MYBPC1, COL1A1, and CHRNB1 were related to musculoskeletal disorders. The sequence alignment of COL1A1, COL6A3, MFN2 MYBPC1 and MYH6 was high (> 80%) in pigs vs. cattle or pigs vs. sheep. Although the difference in the mRNA sequences of CHRNB1 and TNNT1 was high (> 30%), their protein identities were also high. Alignment analysis of these genes revealed a high degree of conservation, providing evidence that these genes may result in the same disorders in pigs (Table 3). Based on the ceRNA network and PPI module 3, CCN2, THBS1, TNNC1 and TPM3 were identified as the key genes. Furthermore, the ISwine [18] platform was used to obtain information about the traits associated with the 19 genes. In the ISwine integration database, Quantitative Trait-associated Locis (QTALs), Quantitative Trait-Associated Genes (QTAGs), and Quantitative Trait Associated Nucleotides (QTANs) are referred to as QTXs. Only the QTXs related to muscle and some traits with economic benefit (disease, fat, growth, meat quality, reproduction, slaughter) were retained. We observed that STIF_L01-1 was a QTL for CNN2 that was related to muscle fiber traits (Table 4).

**Table 2:** The mRNAs associated with Mendelian inheritance disorders

Animal	Gene	Phenotype name
Sus scrofa (pig)	MYH7	Tremor, high-frequency
Sus scrofa (pig)	RYR1	Malignant hyperthermia
Sus scrofa (pig)	FBN1	Marfan syndrome
Sus scrofa (pig)	CFH	Membranoproliferative glomerulonephritis type II
Ovis aries (sheep)	TNNT1	Muscular dystrophy, TNNT1-related
Bos taurus (cattle)	MYH6	Abortion (embryonic lethality), MYH6-related
Bos taurus (cattle)	MYBPC1	Arthrogryposis, distal, type 1B
Bos taurus (cattle)	COL1A1	Osteogenesis imperfecta, type II, COL1A1-related
Bos taurus (cattle)	COL6A3	Lethality, COL6A3-related
Bos taurus (cattle)	CHRNB1	Arthrogryposis multiplex congenital, CHRNB1-related
Bos taurus (cattle)	MFN2	Axonopathy

**Table 3:** Gene conservatism analysis

Name	Type	Identity	Similar residues	Gap
CHRNB1	mRNA	83.95% (2087/2486)	-	35.02% (1340/3826)
	Protein	94.65% (478/505)	4.16% (21/505)	0.00% (0/505)
COL1A1	mRNA	94.18% (5569/5913)	-	5.42% (339/6252)
	Protein	97.33% (1424/1463)	2.60% (38/1463)	2.34% (35/1498)
COL6A3	mRNA	85.07% (8460/9945)	-	7.51% (808/10,753)
	Protein	84.36% (2610/3094)	12.64% (391/3094)	4.57% (148/3242)
MFN2	mRNA	84.89% (3675/4329)	-	11.24% (548/4877)
	Protein	98.68% (747/757)	1.19% (9/757)	0.00% (0/757)
MYBPC1	mRNA	91.13% (3606/3957)	-	15.97% (752/4709)
	Protein	89.39% (1078/1206)	9.29% (112/1206)	3.37% (42/1248)
MYH6	mRNA	94.07% (5472/5817)	-	12.28% (814/6631)
	Protein	95.98% (1860/1938)	3.15% (61/1938)	5.56% (114/2052)
TNNT1	mRNA	77.64% (736/948)	-	52.62% (1053/2001)
	Protein	86.69% (228/263)	7.22% (19/263)	11.74% (35/298)

**Table 4:** Information on CNN2-related QTXs

QTX name	Type	Classify	Trait description
STIF_L01-1	Meat	Stiffening	Muscle fiber traits
MCOL_L01-3	Meat	Meat color	Meat color a*
MCOL_L01-4	Meat	Meat color	Colour a on the quadriceps femoris on the ham
TEXT_N01-8	Meat	Texture	Marbling score
TEXT_L01-2	Meat	Texture	CFAT
WHC_L01-1	Meat	Water holding capacity	MOIST
TEXT_L01-1	Meat	Texture	MARB
PH_L01-2	Meat	pH	Loin pH 1
MCT_L01-1	Muscle	Meat composition trait	Protein percentage in muscle
LMA_L01-1	Muscle	Longissimus thoracis muscle	EMA
FACOM_L01-11	Fat	Fatty acid composition	Stearic acid content
FACOM_L01-12	Fat	Fatty acid composition	Stearic acid content
EXTF_L01-1	Fat	External fat	Carcass traits
BFT_L01-4	Fat	Backfat thickness	Backfat thickness at last rib (cm)
FACOM_L01-7	Fat	Fatty acid composition	Linoleic acid content
FACOM_L01-8	Fat	Fatty acid composition	Palmitic acid content
FACOM_L01-9	Fat	Fatty acid composition	Saturated fatty acid content
FACOM_L01-10	Fat	Fatty acid composition	Unsaturated fatty acid content
BFT_L01-5	Fat	Backfat thickness	Back fat thickness on loin at 13-14th rib (cm)
BFT_L01-2	Fat	Backfat thickness	Shoulder BFT (cm)
BFT_L01-1	Fat	Backfat thickness	Average BFT (cm)
FA_L01-1	Fat	Fat area	Fat area (cm2)
ADG_L01-7	Growth	Average daily gain	Production trait
BW_L01-7	Growth	Body weight	Body weight at 60 day
ADG_L01-1	Growth	Average daily gain	Average daily gain from test start to slaughter (g/day)
LT_L01-1	Reproduction	Litter trait	Number born alive
LT_L01-7	Reproduction	Litter trait	Life growth
OST_L01-2	Slaughter	Organ size trait	Carcass trait
CLEN_L01-1	Slaughter	Carcass length	Carcass traits
CLEN_L01-2	Slaughter	Carcass length	Carcass traits
OST_L01-3	Slaughter	Organ size trait	Carcass traits
OST_L01-4	Slaughter	Organ size trait	Carcass traits
BONE_L01-1	Slaughter	Bone trait	Bone trait
CW_L01-2	Slaughter	Carcass weight	Carcass traits
CW_L01-1	Slaughter	Carcass weight	Carcass traits
DCCT_L01-1	Slaughter	Dressed carcass composition trait	Estimated belly lean content (%)
DCCT_L01-2	Slaughter	Dressed carcass composition trait	Estimated carcass lean content (%)
DCCT_L01-3	Slaughter	Dressed carcass composition trait	Ratio of fat area to meat area (%)

## Discussion

Pork represents a vital source of high-quality protein within the human diet, and enhancing pork quality is a prominent subject in advancing the livestock industry. As essential components of muscles, muscle fibers play a crucial role in determining different muscle types and revealing various characteristics associated with meat quality. Most of the studies on the molecular mechanism of muscle fiber development in pork involve protein-coding genes. In recent years, it has been discovered that ncRNAs play important roles in the regulation of muscle growth and development. LncRNA-FKBP1C regulates muscle fiber type conversion by affecting the stability of the MYH1B protein [24]. However, there is a lack of comprehensive screening of miRNA mediated ceRNA networks that reflect the differences between LD and SOL.

In this study, we showed significant differences by comparing the expression of proteins and mRNAs of different muscle fiber types in LD and SOL. The expression profiles of mRNA and ncRNA in LD and SOL were then compared to identify the key factors affecting the transformation of fast and slow muscle. By integrating the interactions between DEmiRs and DEmRs or DElRs, we constructed a ceRNA network that included 19 miRNAs, 32 mRNAs and 134 lncRNAs. The results of KEGG pathway enrichment analysis revealed that ceRNA-related RNAs were significantly enriched in pathways related to muscle growth and development. The GO enrichment results suggested that the ceRNA network of lncRNA–miRNA–mRNA might regulate the biological processes and pathways related to muscle contraction. In our ceRNA network, the nodes ssc-miR-378, ssc-miR-378b-3p, ssc-miR-24-3p, XR_308817, XR_308823 SMIM8, MAVS, and FOS had higher degrees, we verified the binding ability of ssc-miR-24-3p to both SMIM8 and MAVS mRNA by double luciferase report assays. It was reported that miR-24-3p promotes proliferation and apoptosis in rat and bovine skeletal muscle cells by targeting CAMK2B [25], in addition to studies expressing that miR-24-3p is associated with the promotion of skeletal muscle cell differentiation and regeneration in mice by targeting the HMGA1 gene [19] to large white pigs. Danyang Fan et al. found that miR-24-3p was expressed at a higher level in muscle tissues of Tongcheng (obese-type) pigs and in the longest dorsal muscle, which is the same as our findings [26], they found that miR-24-3p was shown to facilitate the conversion of slow muscle fibers to fast muscle fibers and influence the expression of GLUT4, a glucose transporter. SMIM8 gene has been reported to be associated with loss of insulin sensitivity (IS) and oxidative phosphorylation biological processes in skeletal muscle [27]. Our study identified a new target gene of miR-24-3p, the SMIM8 gene, and we hypothesize that miR-24-3p may regulate myofibre formation or transformation of myofibre types by targeting the SMIM8 gene. Further investigation is required to confirm these hypotheses.

We found that miR-99b and miR-125a were highly expressed in the SOL, while miR-10a-3p and miR-10a-5p were highly expressed in the LD. miR-99b-3p [28] have been reported to affect the proliferation and differentiation of skeletal muscle satellite cells. We found the target genes of the miR-10 family including DES, MYBPH and SMAD3, and found that miR-10a-5p had a strong binding ability with MYBPH through dual luciferase reporter assays. The study demonstrated that MYBPH expression was higher in the Meishan pig LD muscle compared to the flounder muscle [29]. Additionally, the gene was found to be associated with cardiomyocyte contraction [30]. Further investigation is required to determine the involvement of miR-10a-5p in myofibre-type transformation by targeting the MYBPH gene. In general, our study identified several key mirnas and their ceRNA regulatory networks that may be involved in the formation or transformation of muscle fiber types in porcine muscle, and.provide a theoretical basis for pork quality improvement.

## Conclusions

Overall, we compared the differences between LD and SOL, and identified 312 demr, 30 demir, 183 delr, and 3417 decr by transcriptome sequencing of LD and SOL. The ceRNA network revealed that the ssc-miR-378, ssc-miR-378b-3p, ssc-miR-24-3p, XR_308817 XR_308823, SMIM8, MAVS and FOS nodes have an extremely high degree, indicating that these molecules are at the core of the network and play important roles in LD and SOL. We verified that miR-24-3p has the binding ability to SMIM8 and MAVS through dual luciferase reports, and plays an important role in the regulation of muscle fiber types. The PPI network analysis revealed that ACTC1, FN1, ACTG2, ACTN2, HSPA4, HSPA9, HSPCB, HSP90AA1 and ITGB6 had the highest number of nodes according to the sequencing results and were related to fast and slow fiber conversion, skeletal muscle contraction and muscle contraction. In addition, miR-125a and miR-24-3p were confirmed to have significantly greater expression in SOL than in LD, while miR-378 and miR-10a-5p had significantly greater expression in LD than in SOL. We found the target genes of the miR-10 family including DES, MYBPH and SMAD3 through the ceRNA network, and found that miR-10a-5p had a strong binding ability with MYBPH through dual luciferase. Therefore, it can be concluded that this gene plays a key role in the regulation of meat quality and muscle fiber type conversion.

### Electronic supplementary material

Below is the link to the electronic supplementary material.

Supplementary Material 1

## Data Availability

The raw sequence data reported in this paper have been deposited in the Genome Sequence Archive in China National Center for Bioinformation (CRA015020) that are accessible at https://ngdc.cncb.ac.cn/gsab/. Please refer to the attachment for the processed data files.

## Abbreviations

LD: Longissimus dorsi
SOL: Soleus
miRNA: MicroRNAs
lncRNA: Long noncoding RNAs
circRNA: Circular RNAs
ceRNA: Competitive endogenous RNA
PGC-1α: Peroxisome proliferator-activated receptor gamma coactivator 1α
DEmRs: Differentially expressed mRNAs
DEmiRs: Differentially expressed miRNAs
DElRs: Differentially expressed lncRNAs
DEcRs: Differentially expressed circRNAs
GO: Gene Ontology
KEGG: Kyoto Encyclopedia of Genes and Genomes
PPI: Protein‒protein interaction
MCODE: Molecular Complex Detection
OMIA: Online Mendelian Inheritance in Animals
QTALs: Quantitative Trait-associated Locis
QTAGs: Quantitative Trait-Associated Genes
QTANs: Quantitative Trait Associated Nucleotides
GWASs: Genome-Wide Association Studies
WT: wild-type
MUT: mutantion

## References

[1] Currie RW, Wolfe FH (1977). Evidence for differences in post mortem intramuscular phospholipase activity in several muscle types. Meat Sci.

[2] Zhang L, Zhou Y, Wu W, Hou L, Chen H, Zuo B, Xiong Y, Yang J (2017). Skeletal muscle-specific overexpression of PGC-1α induces Fiber-type Conversion through enhanced mitochondrial respiration and fatty acid oxidation in mice and pigs. Int J Biol Sci.

[3] Zhang S, Chen X, Huang Z, Chen D, Yu B, Chen H, He J, Luo J, Zheng P, Yu J (2019). Leucine promotes porcine myofibre type transformation from fast-twitch to slow-twitch through the protein kinase B (akt)/forkhead box 1 signalling pathway and microRNA-27a. Br J Nutr.

[4] Cao H, Liu J, Du T, Liu Y, Zhang X, Guo Y, Wang J, Zhou X, Li X, Yang G (2022). Circular RNA screening identifies circMYLK4 as a regulator of fast/slow myofibers in porcine skeletal muscles. Mol Genet Genomics: MGG.

[5] Ju X, Liu Y, Shan Y, Ji G, Zhang M, Tu Y, Zou J, Chen X, Geng Z, Shu J (2021). Analysis of potential regulatory LncRNAs and CircRNAs in the oxidative myofiber and glycolytic myofiber of chickens. Sci Rep.

[6] Dou M, Yao Y, Ma L, Wang X, Shi X, Yang G, Li X (2020). The long noncoding RNA MyHC IIA/X-AS contributes to skeletal muscle myogenesis and maintains the fast fiber phenotype. J Biol Chem.

[7] Sun J, Xie M, Huang Z, Li H, Chen T, Sun R, Wang J, Xi Q, Wu T, Zhang Y (2017). Integrated analysis of non-coding RNA and mRNA expression profiles of 2 pig breeds differing in muscle traits. J Anim Sci.

[8] Zhong Z, Huang M, Lv M, He Y, Duan C, Zhang L, Chen J (2017). Circular RNA MYLK as a competing endogenous RNA promotes bladder cancer progression through modulating VEGFA/VEGFR2 signaling pathway. Cancer Lett.

[9] Cao H, Du T, Li C, Wu L, Liu J, Guo Y, Li X, Yang G, Jin J, Shi X (2023). MicroRNA-668-3p inhibits myoblast proliferation and differentiation by targeting Appl1. BMC Genomics.

[10] Kertesz M, Iovino N, Unnerstall U, Gaul U, Segal E (2007). The role of site accessibility in microRNA target recognition. Nat Genet.

[11] Miranda KC, Huynh T, Tay Y, Ang YS, Tam WL, Thomson AM, Lim B, Rigoutsos I (2006). A pattern-based method for the identification of MicroRNA binding sites and their corresponding heteroduplexes. Cell.

[12] Friedman RC, Farh KK, Burge CB, Bartel DP (2009). Most mammalian mRNAs are conserved targets of microRNAs. Genome Res.

[13] Smoot ME, Ono K, Ruscheinski J, Wang PL, Ideker T (2011). Cytoscape 2.8: new features for data integration and network visualization. Bioinf (Oxford England).

[14] Sherman BT, Hao M, Qiu J, Jiao X, Baseler MW, Lane HC, Imamichi T, Chang W (2022). DAVID: a web server for functional enrichment analysis and functional annotation of gene lists (2021 update). Nucleic Acids Res.

[15] Hu H, Miao YR, Jia LH, Yu QY, Zhang Q, Guo AY (2019). AnimalTFDB 3.0: a comprehensive resource for annotation and prediction of animal transcription factors. Nucleic Acids Res.

[16] Altaf-Ul-Amin M, Shinbo Y, Mihara K, Kurokawa K, Kanaya S (2006). Development and implementation of an algorithm for detection of protein complexes in large interaction networks. BMC Bioinformatics.

[17] Lenffer J, Nicholas FW, Castle K, Rao A, Gregory S, Poidinger M, Mailman MD, Ranganathan S (2006). OMIA (Online mendelian inheritance in animals): an enhanced platform and integration into the Entrez search interface at NCBI. Nucleic Acids Res.

[18] Fu Y, Xu J, Tang Z, Wang L, Yin D, Fan Y, Zhang D, Deng F, Zhang Y, Zhang H (2020). A gene prioritization method based on a swine multi-omics knowledgebase and a deep learning model. Commun Biology.

[19] Dey P, Soyer MA, Dey BK (2022). MicroRNA-24-3p promotes skeletal muscle differentiation and regeneration by regulating HMGA1. Cell Mol Life Sci.

[20] Richardson E, Bohrer BM, Arkfeld EK, Boler DD, Dilger AC (2017). A comparison of intact and degraded desmin in cooked and uncooked pork longissimus thoracis and their relationship to pork quality. Meat Sci.

[21] López-Pedrouso M, Lorenzo JM, Cittadini A, Sarries MV, Gagaoua M, Franco D (2023). A proteomic approach to identify biomarkers of foal meat quality: a focus on tenderness, color and intramuscular fat traits. Food Chem.

[22] He N, Lang X, Wang C, Lv C, Li M, Sun R, Zhang J (2023). Expression of MSTN/Smad signaling pathway genes and its association with meat quality in tibetan sheep (Ovis aries). Food Sci Nutr.

[23] Jia X, Wu J, Chen X, Hou S, Li Y, Zhao L, Zhu Y, Li Z, Deng C, Su W (2023). Cell atlas of trabecular meshwork in glaucomatous non-human primates and DEGs related to tissue contract based on single-cell transcriptomics. iScience.

[24] Yu JA, Wang Z, Yang X, Ma M, Li Z, Nie Q (2021). LncRNA-FKBP1C regulates muscle fiber type switching by affecting the stability of MYH1B. Cell Death Discovery.

[25] Yang G, Wu M, Liu X, Wang F, Li M, An X, Bai F, Lei C, Dang R. MiR-24-3p conservatively regulates muscle cell proliferation and apoptosis by Targeting Common Gene CAMK2B in rat and cattle. Animals: Open Access J MDPI 2022, 12(4). 10.3390/ani12040505 PMC8868287 35203213

[26] Fan D, Yao Y, Liu Y, Yan C, Li F, Wang S, Yu M, Xie B, Tang Z. Regulation of myo-mir-24-3p on the Myogenesis and Fiber Type Transformation of skeletal muscle. Genes 2024, 15(3). 10.3390/genes15030269 PMC10970682 38540328

[27] Timmons JA, Atherton PJ, Larsson O, Sood S, Blokhin IO, Brogan RJ, Volmar CH, Josse AR, Slentz C, Wahlestedt C (2018). A coding and non-coding transcriptomic perspective on the genomics of human metabolic disease. Nucleic Acids Res.

[28] Liao R, Lv Y, Dai J, Zhang D, Zhu L, Lin Y. chi-miR-99b-3p Regulates the Proliferation of Goat Skeletal Muscle Satellite Cells In Vitro by Targeting Caspase-3 and NCOR1. *Animals: an open access journal from MDPI* 2022, 12(18). 10.3390/ani12182368 PMC9495177 36139227

[29] Li Y, Xu Z, Li H, Xiong Y, Zuo B (2010). Differential transcriptional analysis between red and white skeletal muscle of Chinese Meishan pigs. Int J Biol Sci.

[30] Mouton J, Loos B, Moolman-Smook JC, Kinnear CJ (2015). Ascribing novel functions to the sarcomeric protein, myosin binding protein H (MyBPH) in cardiac sarcomere contraction. Exp Cell Res.

