# Supplementary material for: Identification of different myofiber types in pigs muscles and construction of regulatory networks
Source: BMC Genomics. 2024 Apr 24;25:400. doi: 10.1186/s12864-024-10271-9 (PMC11040794; doi:10.1186/s12864-024-10271-9)

**Gel images original**

**Fig. S1**


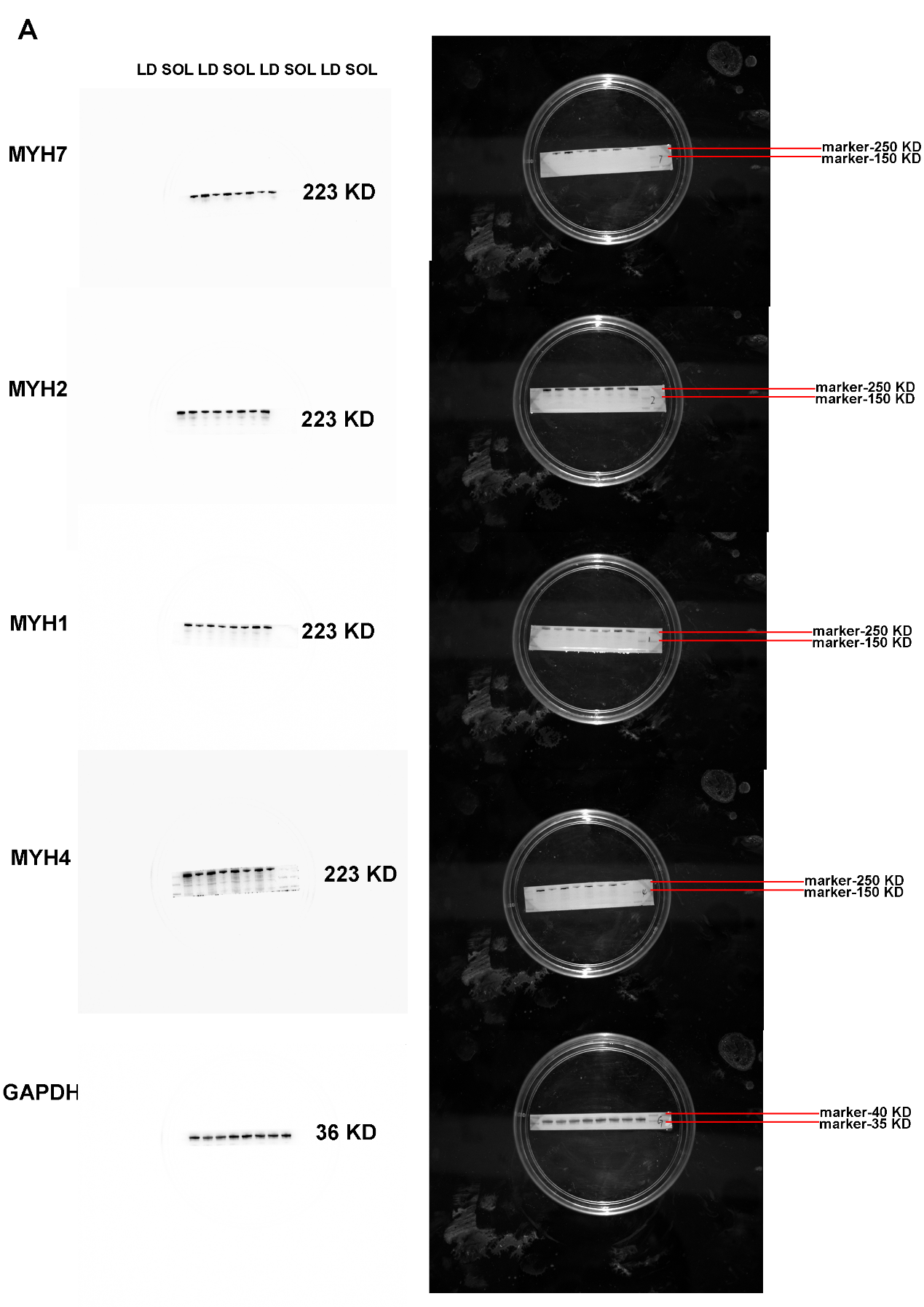


**Fig. S1: Identification of muscle protein differences in LD and SOL. A.** Protein levels of MYH7, MYH2, MYH1, and MYH4 as measured via Western blotting n LD and SOL muscles (*n* = 4). (During the blotting process, the polyvinylidene fluoride membrane is sheared before hybridization with the antibody.)

**MYH7**


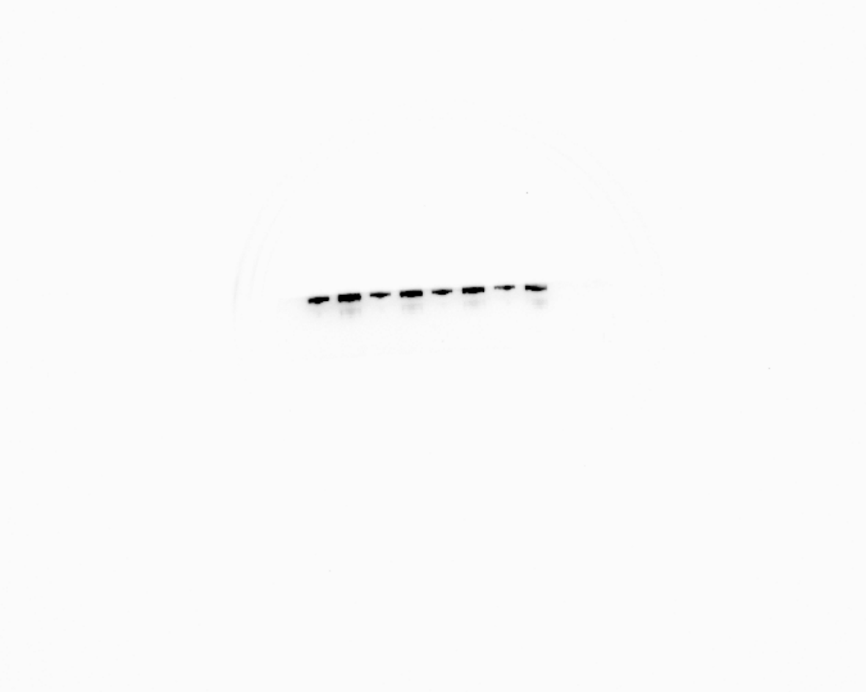


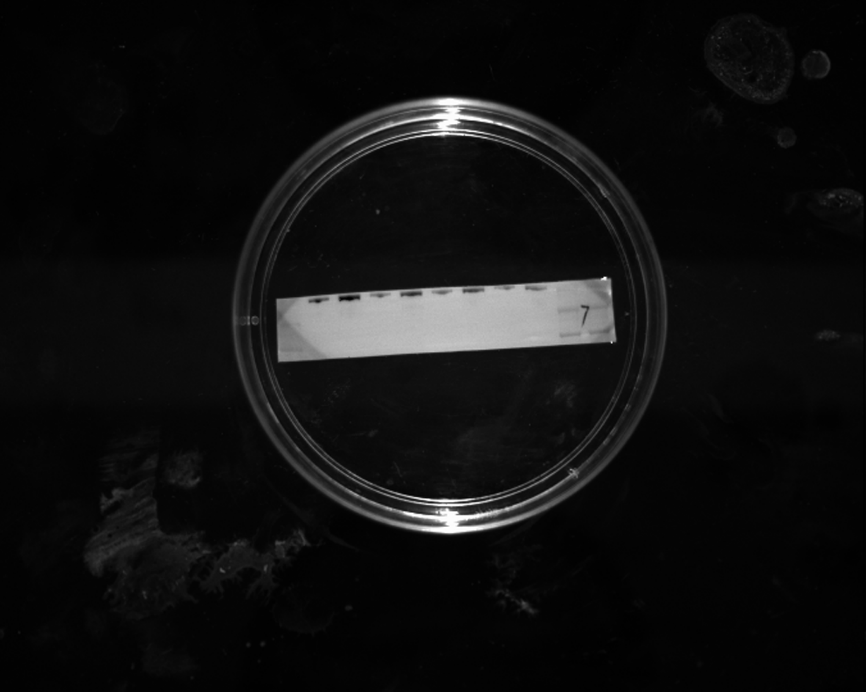


**MYH2**


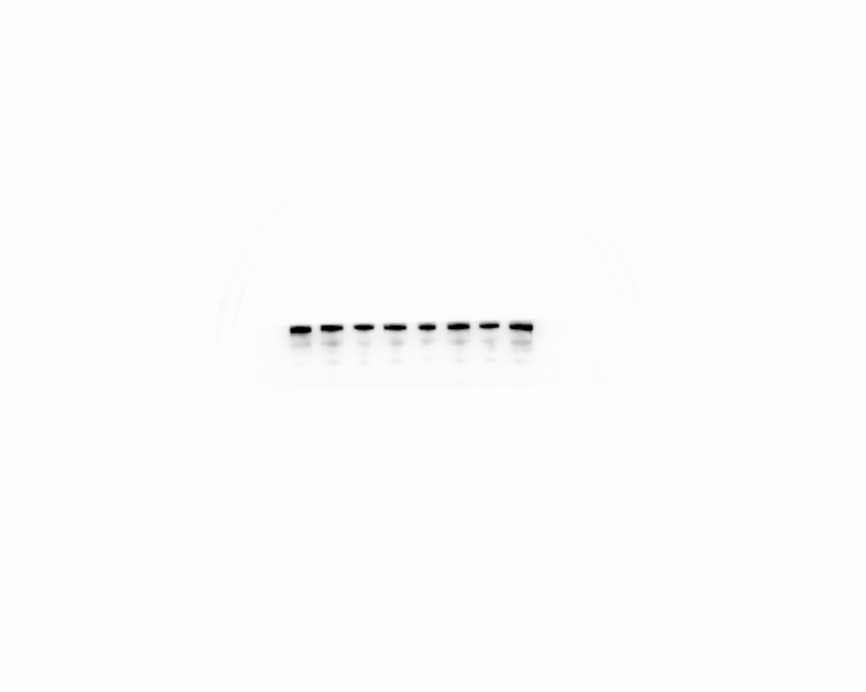


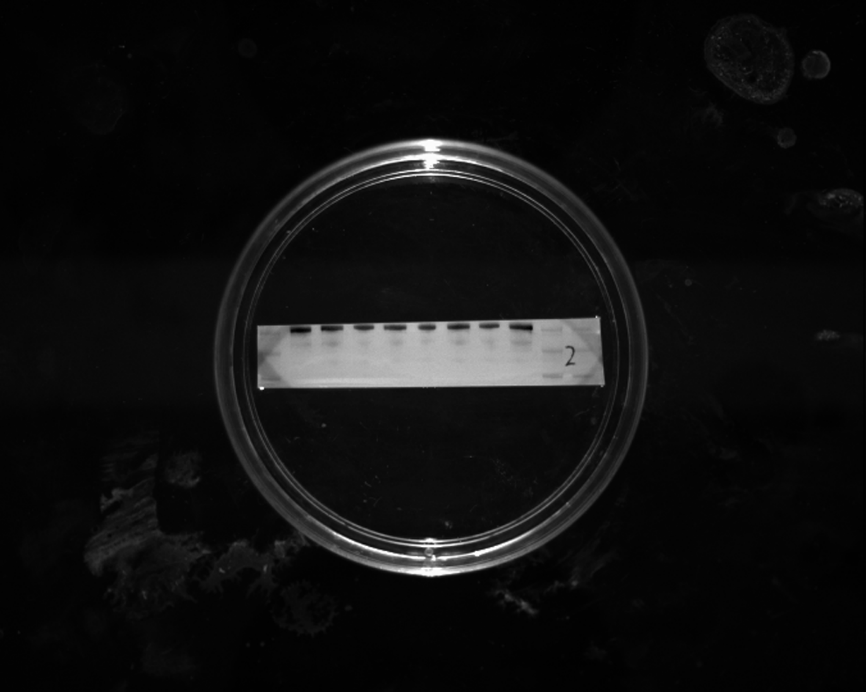


**MYH1**


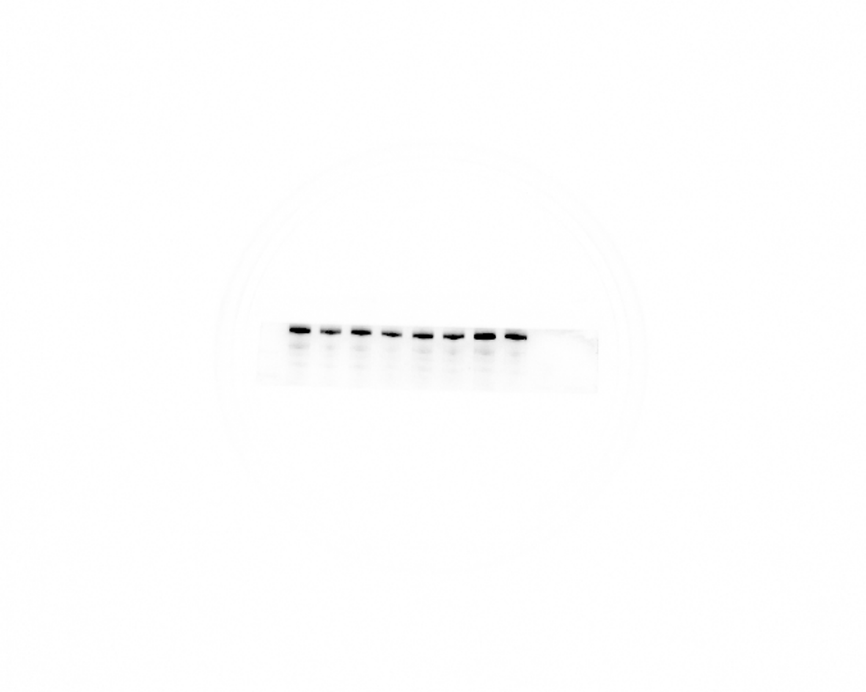


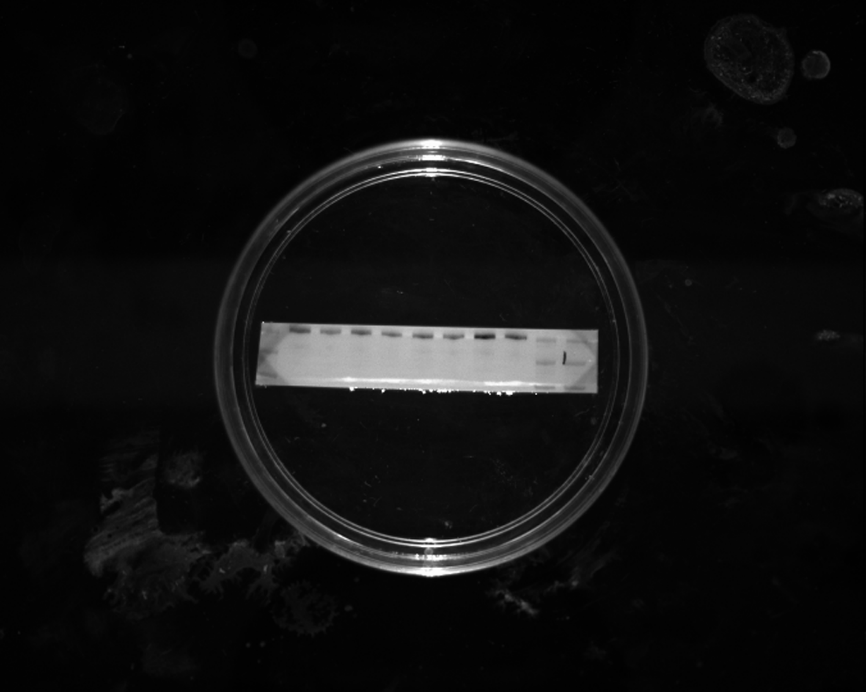


**MYH4**


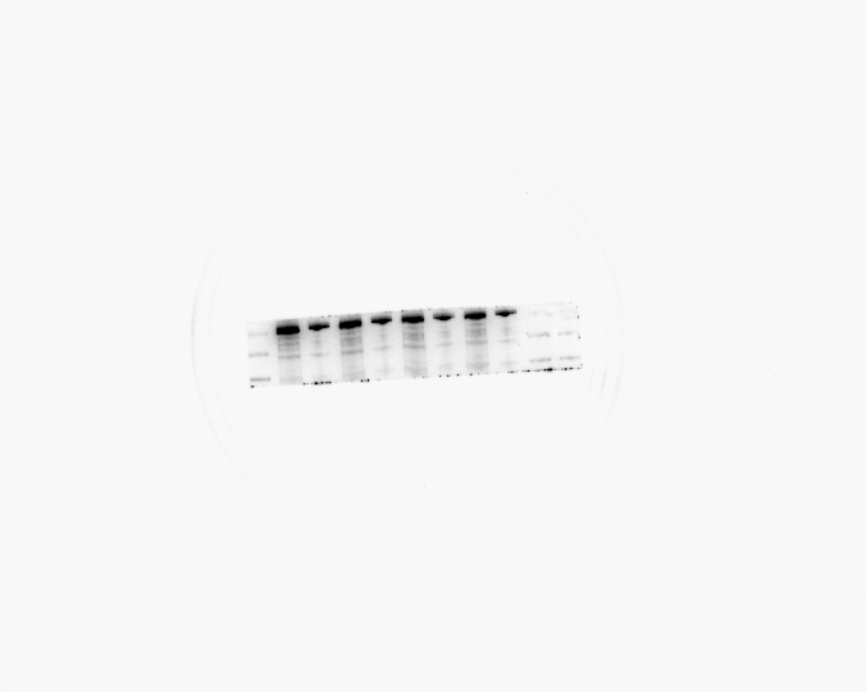


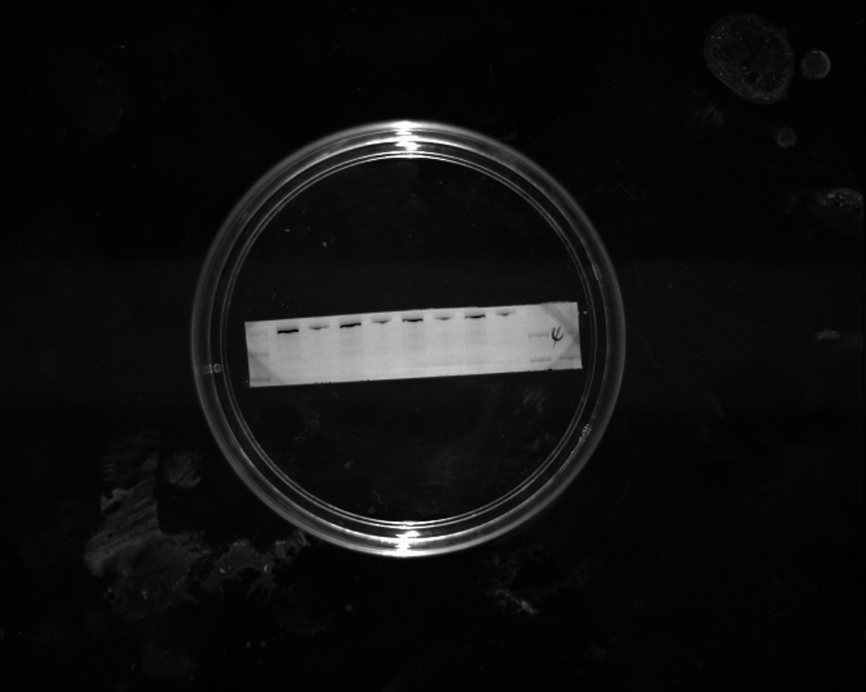


**GAPDH**


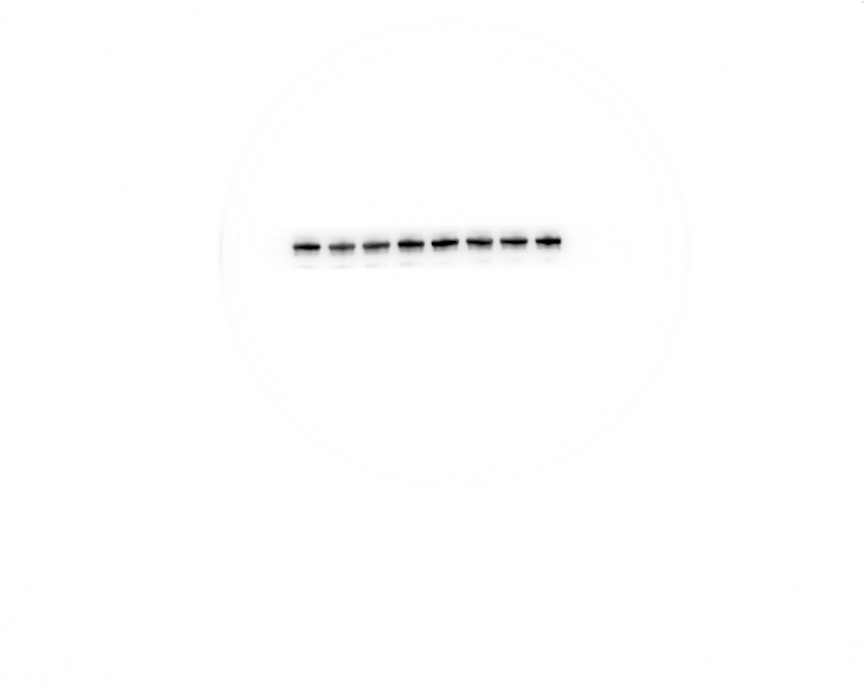


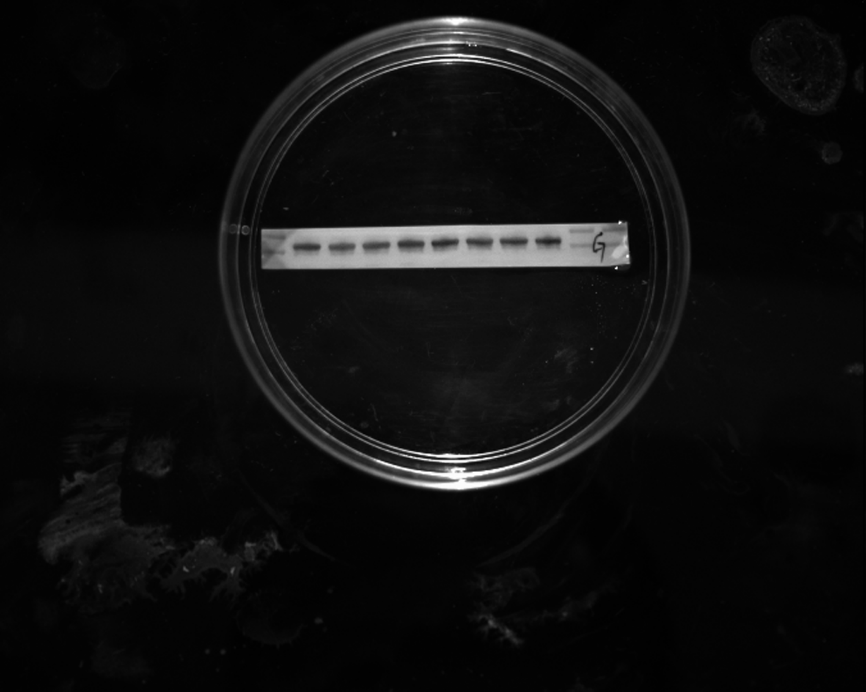

Supplement: Supplementary file 1 — Supplementary Material 1 [file 12864_2024_10271_MOESM1_ESM.docx]
